# Invasive thymoma with intravascular growth into the great veins and right atrium: A case report

**DOI:** 10.1111/1759-7714.13242

**Published:** 2020-03-17

**Authors:** Wang Shen, Yang Cao, Xinyun Wang, Peng Zhang, Qinghua Zhou

**Affiliations:** ^1^ Lung Cancer Center West China Hospital, Sichuan University Chengdu China; ^2^ Tianjin Medical University General Hospital Tianjin China

**Keywords:** Brachiocephalic vein, right atrium, superior vena cava, thymoma

## Abstract

Here, we present a case of invasive thymoma with intraluminal growth into the left and right brachiocephalic veins, superior vena cava and the right atrium. Resection of the mass, double partial upper lobe lobectomy and superior vena cava, and left and right brachiocephalic vein replacement were performed. Pathological diagnosis indicated a mixed histological pattern indicative of type B1 and type B2 thymoma, predominantly type B2 thymoma. The patient was well and without local recurrence or distal metastasis after 50 months of follow‐up.

## Introduction

Thymoma is the most common primary anterior mediastinal tumor in adults, and the annual incidence reported is 0.17 per 100 000 population in China.^1^ Although benign in some cases, it has malignant potential due to local invasion and rare distant metastasis. The intracardiac growth of thymoma is rare. Here, we describe a case of an invasive thymoma presenting as superior vena cava syndrome (SVCS) which was caused by an intraluminal tumor involving the left brachiocephalic vein (LBCV), right brachiocephalic vein (RBCV), superior vena cava (SVC) and the right atrium.

## Case report

**Figure 1:**
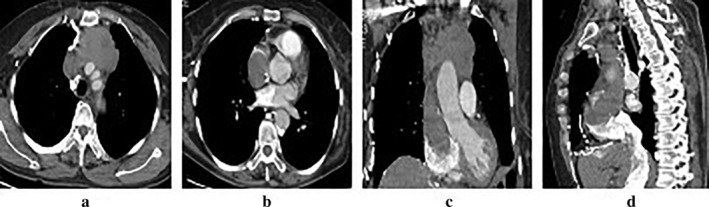
Computed tomographic (CT) scan with intravenous contrast administration. (**a**–**b**) Axial views: A large irregular anterior mediastinal mass with heterogeneous enhancement was seen to obstruct the SVC and had extended into the right atrium. (**c**–**d**) Coronal and sagittal views: the mass located in the anterior mediastinum encased the mediastinal great vessels, and infiltrated into the right atrium.

A 63‐year‐old Chinese female was admitted to our clinic with progressive exertional dyspnea, and upper limb and facial edema of 60 days duration. There were no clinical signs of myasthenia gravis. An enhanced chest computed tomography (CT) scan revealed a 77 mm × 67 mm irregular anterior mediastinal mass with heterogeneous enhancement compressing the mediastinal great vessels, invading the LBCV and growing into the right atrium, associated with intraluminal filling defects. Multiple collateral vessels were seen in the right anterior chest wall (Fig 1). Fine needle aspiration (FNA) was carried out and revealed a diagnosis of thymoma.

**Figure 2:**
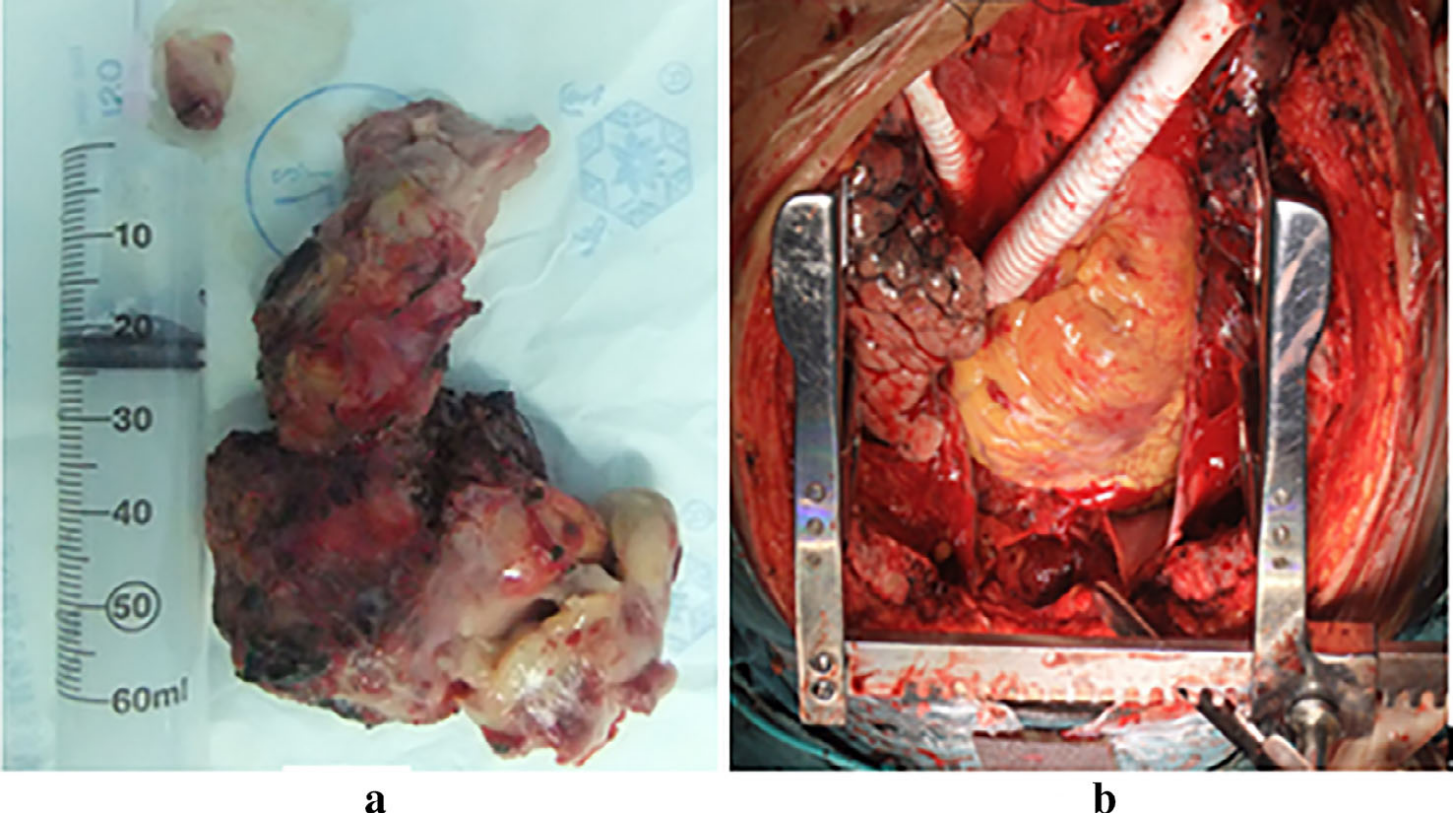
(**a**) The tumor was a huge grayish‐yellow tough mass, measuring 12 cm × 8.5 cm × 7 cm. (**b**) The left and right brachiocephalic veins were replaced with grafts.

**Figure 3:**
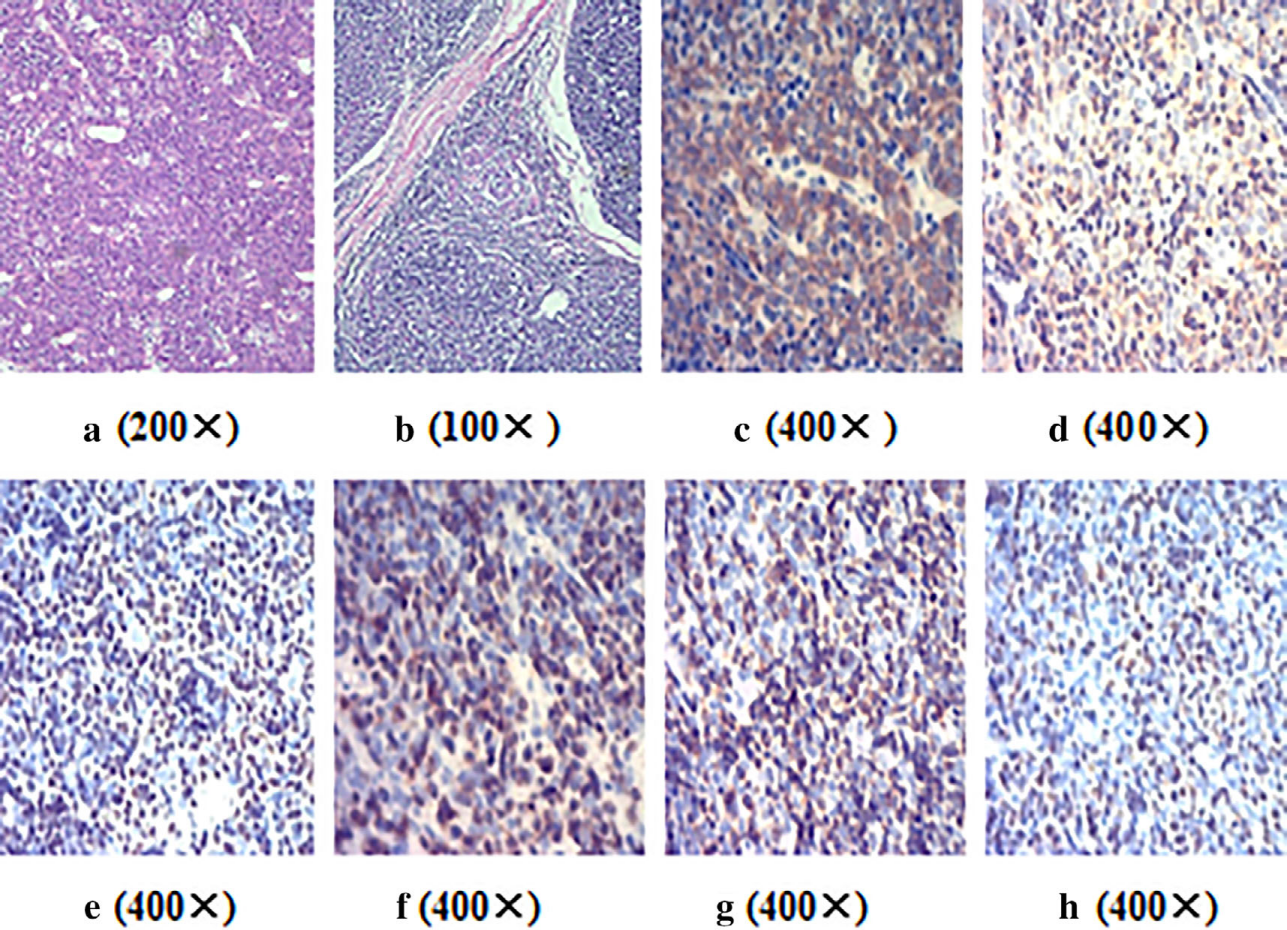
(**a**) On microscopic visualization, the tumour had a highly vascular appearance, magnification 200x. (**b**) Some areas were type B1 thymoma (lymphocyte‐rich thymoma), and a few neoplastic epithelial cells could be seen against a background of lymphocytes, magnification 100x. (**c**) CK19 was positive for epithelial cells, magnification 400x. (**d**) CD99 was positive for lymphocytes, magnification 400x. (**e**) TdT was positive for lymphocytes, magnification 400x. (**f**) CD5 was positive for lymphocytes, magnification 400x. (**g**) CD3 was positive for lymphocytes, magnification 400x. (**h**) Ki67 was positive for lymphocytes, magnification 400x.

The patient underwent a radical excision of the mediastinal mass, the invaded parts of the mediastinal pleura and the pericardium, double partial upper lobes, SVC, LBCV and RBCV, with reconstructions of LBCV and RBCV to the right atrium with grafts in August 2011. Intraoperatively, the mass was located in the superior anterior mediastinum and encased the LBCV. It invaded into the right atrium through the LBCV, RBCV and SVC and formed tumor thrombi. Pathologically, the tumor was described as a huge grayish yellow tough mass of 12 cm × 8.5 cm × 7 cm in size (Fig 2). Microscopically, the tumor consisted of neoplastic epithelial cells and non‐neoplastic lymphocytes. Most areas were type B2 thymoma, epithelial cells were arranged in sheets or cords, and some lymphocytes could be seen between tumor cells (Fig 3). The tumour had a highly vascular appearance. Some areas were type B1 thymoma (lymphocyte‐rich thymoma), and a few neoplastic epithelial cells could be seen against a background of lymphocytes. Immunohistochemical staining showed that CK19 was positive for epithelial cells. CD99, TdT, CD5, CD3 and Ki67 were positive for lymphocytes. Histopathological diagnosis indicated a mixed pattern of type B1 and type B2 thymoma, predominantly type B2 thymoma (WHO classification). The tumor stage was IVA due to the pleural and pericardial dissemination (Masaoka stage). The patient was discharged from hospital on postoperative Day 18 and without local recurrence or distant metastasis after 50 months of follow‐up.

## Discussion

Invasive thymoma is characterized by infiltration and extension into the neighboring structures, and metastasis is usually confined to the pleura, pericardium, or diaphragm.^2^ The intraluminal extension of the thymoma with thrombus formation through LBCV, RBCV and SVC into the right atrium is uncommon. In this case, we found that the thymoma was firmly adherent to the vessel intima, and infiltrated the brachiocephalic veins and grew along the venous flow within the SVC to the right atrium.

Patients with thymomas present with various clinical symptoms including cough, chest pain, dyspnea and some autoimmune disorders, especially myasthenia gravis,^3^, ^4^, ^5^ which is present in 30% to 50% of patients with thymoma.^6^ Thymomas leading to SVCS occurred in only approximately 4% of cases in the report by Kallás *et al*., and the most common cause was extrinsic compression.^7^ The intracardiac growth of thymoma is rare.^8^

Histopathologically, the main characteristics of thymoma consist of a wide range of cytologic patterns within thymic epithelial cells, and the association with a nontumoral lymphocytic component, whose relative proportion to neoplastic cells is the basis of the current World Health Organization (WHO) histopathological classification. It is difficult to differentiate thymoma from thymic carcinoid and thymic carcinoma. The presentation of thymoma and thymic carcinoid are very similar; however, lymphocytes are rarely seen in the latter. Thymoma is generally considered cytologically benign, whereas thymic carcinoma has malignant cytologic features. Furthermore, lymphocyte‐rich thymoma can be differentiated from non‐Hodgkin's lymphoma because thymoma consists of neoplastic epithelium cells and non‐neoplastic T‐lymphocytes.

Radiologically, a thymoma characteristically presents as a well‐circumscribed, lobulated or rounded, homogeneous or heterogeneous mass which frequently appears to touch the sternum. While subtle invasion may be difficult to detect, an infiltrative appearance, obvious vascular compromise, and/or an irregular interface with adjacent structures are highly suggestive findings of invasion.

In summary, synchronous thrombus of the superior vena cava and/or brachiocephalic veins and mediastinal thymoma in this report indicated the possibility of invasive thymoma intraluminal extension with thrombus formation. Moreover, in the rare event of an intravascular thymoma growth, a radical excision of the tumor and replacement of invaded great vessels with grafts can achieve a good outcome.
